# Prediction of DNA i-motifs via machine learning

**DOI:** 10.1093/nar/gkae092

**Published:** 2024-02-14

**Authors:** Bibo Yang, Dilek Guneri, Haopeng Yu, Elisé P Wright, Wenqian Chen, Zoë A E Waller, Yiliang Ding

**Affiliations:** Department of Cell and Developmental Biology, John Innes Centre, Norwich Research Park, Norwich NR4 7UH, UK; School of Pharmacy, University College London, London WC1N 1AX, UK; Department of Cell and Developmental Biology, John Innes Centre, Norwich Research Park, Norwich NR4 7UH, UK; Molecular Physiology School of Medicine, and Molecular Medicine Research Group, University of Western Sydney, Campbelltown, NSW 1797, Australia; School of Pharmacy, University College London, London WC1N 1AX, UK; School of Pharmacy, University College London, London WC1N 1AX, UK; Department of Cell and Developmental Biology, John Innes Centre, Norwich Research Park, Norwich NR4 7UH, UK

## Abstract

i-Motifs (iMs), are secondary structures formed in cytosine-rich DNA sequences and are involved in multiple functions in the genome. Although putative iM forming sequences are widely distributed in the human genome, the folding status and strength of putative iMs vary dramatically. Much previous research on iM has focused on assessing the iM folding properties using biophysical experiments. However, there are no dedicated computational tools for predicting the folding status and strength of iM structures. Here, we introduce a machine learning pipeline, iM-Seeker, to predict both folding status and structural stability of DNA iMs. The programme iM-Seeker incorporates a Balanced Random Forest classifier trained on genome-wide iMab antibody-based CUT&Tag sequencing data to predict the folding status and an Extreme Gradient Boosting regressor to estimate the folding strength according to both literature biophysical data and our in-house biophysical experiments. iM-Seeker predicts DNA iM folding status with a classification accuracy of 81% and estimates the folding strength with coefficient of determination (*R*^2^) of 0.642 on the test set. Model interpretation confirms that the nucleotide composition of the C-rich sequence significantly affects iM stability, with a positive correlation with sequences containing cytosine and thymine and a negative correlation with guanine and adenine.

## Graphical Abstract

**Graphical Abstract:**
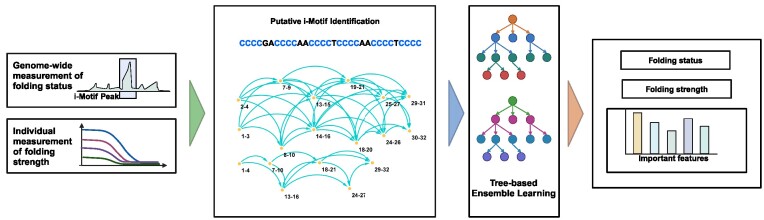

## Introduction

Nucleotides are the basic units that form DNA and RNA, two key molecules in the central dogma. DNA encodes genetic information, which is transcribed to mRNA and then translated to protein. In addition to this transfer of information, DNA and RNA can form complex structures, which can play crucial functional roles in organisms. Besides the canonical Watson-Crick double-helical B-form structure, DNA can form non-canonical secondary structures such as G-quadruplexes (G4s) and i-Motifs (iMs). G4s are four-stranded structures formed from G-rich sequences and are stabilised by Hoogsteen hydrogen bonding between guanines (1). iMs are also four-stranded structures, but formed from cytosine C-rich regions that are stabilised by hemi-protonated C-C base pairs (C^+^:C) (2,3). Complementary G-rich and C-rich sequences can form G4s and iMs interdependently during distinct cellular processes (4). As a non-canonical structure, iMs are indicated to play an important role in the genome. There are an increasing number of *in vitro* and *in celluo* studies that report evidence that iMs could fold in promotor region of certain genes, telomeres and untranslated regions. They have also been implicated as a regulatory element associated with the cell cycle, transcription, chromatin remodelling, as well as transposable element dynamics (5–7).

Commonly, computational analysis of putative iMs is limited to indirect identification by searching for potential complementary G4 sequences in the genome (8). Plenty of G4 prediction tools have been developed previously, and these can generally be divided into two categories based on whether or not the models utilised experimentally derived G4-specific data. Classical computational tools which do not use G4-specific data, are typically constructed from string-matching models based on a specific sequence pattern. Others use a designed scoring system according to pre-defined rules. For example, platforms like Quadparser (9), Quadruplexes (10), AllQuads (11) and QuadBase2 (12) used algorithms like regular expression to search G4 forming sequences, whilst QGRS Mapper (13), G4P (14), and G4Hunter (15), use scoring models that can estimate the probability or strength of putative G4s (16). These models have potential to be used in iM-forming sequences searching, because the putative iMs have in principle similar sequence patterns and some of the rules will be transferrable to both structures. For example, enrichment of G/C in a C/G-rich sequence disfavours both G4 and iMs. In contrast, there are also platforms guided by G4-specific data (e.g. biophysical properties, G4 Chip-seq, G4 CUT&Tag, and G4-seq) that can capture additional G4-specific features to improve the G4 prediction performance (17). Software like PQSfinder (18), G4boost (19), Quadron (20), DeepG4 (21), and G4-folding energy estimation module integrated in RNAFold (22) use data from G4-specific experiments to increase the accuracy of predictions with multiple modeling strategies (e.g. G4Boost and Quadron used ensemble learning and DeepG4 applied deep convolutional neural network(19–21)). Therefore, the application of these models on iM identification is limited. Out of the existing searching platforms, G4Hunter is the easiest to use for searching for iMs as it was designed to take into account C with negative values both to disfavour regions rich in alternative G/C and to score both strand of a DNA duplex simultaneously. C-richness and C-skew is obviously important for iM formation (15). G4-iM Grinder can also be used to predict and evaluate G4 and iM forming sequences (23). G4-iM Grinder was originally designed for identifying G-quadruplexes. Its standout feature is the remarkable flexibility it offers in defining G-quadruplex structures, coupled with the integration of various scoring systems, such as G4Hunter (15), PQSfinder (18) and cGcC (24). However, the G4-iM Grinder scoring system was designed for G-quadruplexes, not i-motif. Based on the distinguished biophysical properties between G-quadruplexes and i-motifs, it is important to have a specific i-motif searching software that cooperates with experimental data. Typically, individual C-rich sequences are biophysically assed for their capability to form iMs. UV spectroscopy is typically used to determine the thermodynamic properties such as melting (*T*_M_) and annealing (*T*_A_) temperatures (25). Furthermore, thermal difference spectra (TDS) are typically generated, using the difference in absorbance spectra between folded and unfolded DNA, determining a signature to identify the formed secondary DNA structure (26). UV spectroscopy is often accompanied with circular dichroism (CD) spectroscopy to confirm the formation of i-motif structure. The transitional pH (pH_T_) is an important measure of the stability of iM structures, determined by assessing the formation of iM across a pH-range (8,27–29).

A systematic prediction tool to identify DNA iM folding status and their potential stability is lacking. Recently, the landscape of iM forming sequences in the whole human genome was determined via the novel CUT&Tag sequencing using anti-iM iMab antibodies on living human cells (7). Here we introduce, iM-Seeker, a novel computational pipeline using the genome-wide iM profile (7), iM-stability data from the literature, and our in-house biophysical analysis to predict iM structure formation and stability. iM-Seeker utilised a newly-designed graph-based algorithm to search for putative iM forming sequences within an entered DNA sequence. The Balanced Random Forest script is trained on the iMs identified in the human genome derived from iMab-based CUT&Tag sequencing data (7) and was further developed to predict iM structure folding status within DNA sequences. iM-Seeker also incorporates the Extreme Gradient Boosting (XGBoost) regressor to predict the structure stability, by cross referencing iM forming DNA sequences to their corresponding pH_T_ values. Furthermore, this computational model has shed new insight into the importance of nucleotide composition in iM stability. A positive correlation was observed for sequences containing cytosine and thymine whilst sequences rich in guanine and adenine were found to have a negative correlation with iM stability. Alongside nucleotide composition, long C-tract lengths accompanied with short loop lengths contribute towards high stability of iM structure.

## Materials and methods

### Data collection

We collected the published CUT&Tag sequencing data in the human genome (7). The data was downloaded from the NCBI GEO database (accession number GSE220882). The BigWig format data included iM forming sequences from both 93T449 (WDLPS) cell line and human embryonic kidney (HEK293T) cell line with three biological replicates for each cell line. The focus was concentrated on HEK293T cell data which was presented with more high-confident iM regions than WDLPS cells (7). The downloaded BigWig files were converted to bedGraph files and iM-peak region were cumulated with SEACR v1.3 set to ‘0.01 non stringent’ parameters (7,30). The intersected iM-peak regions among three biological replicates were defined as the final high-confident iM-peak regions. Literature-derived data of i-motif forming sequences and their corresponding pH_T_ values were collected (Supplementary Table S1).

### Graph-based putative i-motif searching

**Figure 1.**
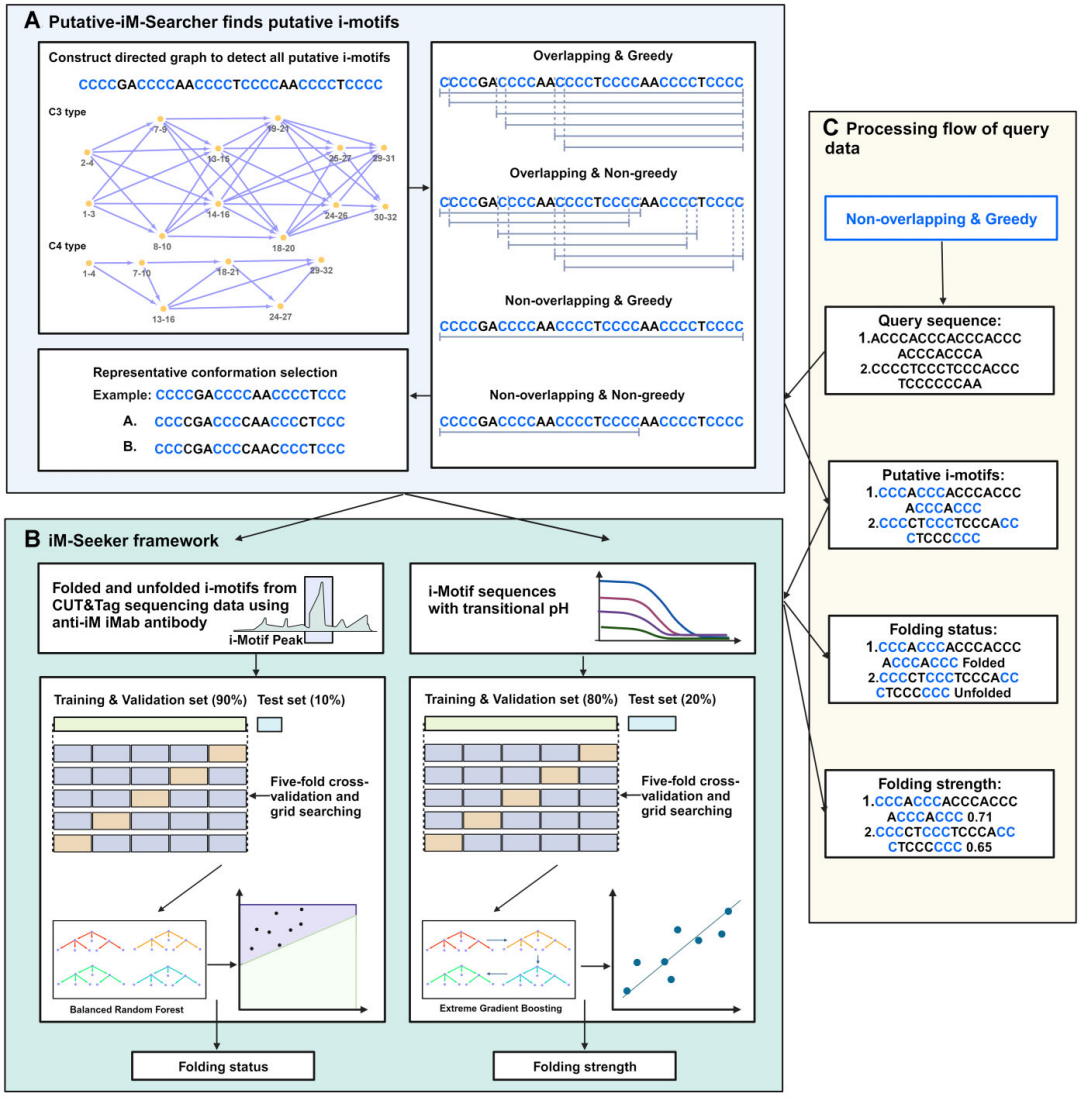
The outline of the whole iM-Seeker. (**A**) The framework of Putative-iM-Searcher. Putative-iM-Searcher can detect all i-motif conformation and representative conformation based on overlapping & non-overlapping strategy, greedy & non-greedy strategy, and representative-conformation strategy. (**B**) The framework of iM-Seeker. iM-Seeker employs Putative-iM-Searcher to find putative i-motif forming sequences on human genome with corresponding published CUT&Tag sequencing data for determining folded and unfolded iM forming sequences and iM-containing sequence with corresponding pH_T_, respectively. For both folding status and strength prediction tasks, the whole dataset was randomly divided into a training & validation set (90% data for folding status prediction task and 80% data for folding strength prediction task, respectively) and a test set (10% data for folding status prediction task and 20% data for folding strength prediction task, respectively). Five-fold cross-validation and grid searching on training & validation set are employed to search for the best hyperparameters and test set is used to evaluate the model's performance. The Balanced Random Forest classification model and XGBoost regression model are trained to predict the folding status and folding strength, respectively. (**C**) The processing flow of query data using iM-Seeker. Created with BioRender.com.

Putative i-motifs can be identified based on their sequence pattern (C_≥3_N_1–12_)_3_C_≥3_ where C represents cytosine and N represent any nucleotide (31,32). The classic approach to identify potential putative iM-forming sequences is to search complementary sequences of G4-forming sequences based on sequence pattern matching. This assumption and current approaches limit the identification of iMs with their different variations in C^+^:C formations and topologies compared to G4s (31,32). For example, many iM sequences are not actually simple ones with only 4 sets of C-tracts. If there are 5 or more sets of C-tracts with different lengths, then it is a question of which 4 of them form the core C^+^:C base pairs within the i-motif structure. For example, the sequence that matches the greedy regular expression rule (C_≥3_N_1–12_)_3_C_≥3_, ‘CCCACCCACCCACCCACCC’, contains 5 sets of C-tracts, then there are 3 possible i-motifs (numbering the C-tracts according to 1–5, the three possibilities are 1235; 1345; 1245). The options are further complicated if the length of the C-tract is different. For example, the sequence matches the non-greedy regular expression rule (C_≥3_N_1–12_)_3_C_≥3_, ‘CCCCACCCACCCCCACCC’, which contains four sets of c-tracts but with different lengths of C. Thus, this simple sequence contains six potential i-motifs, (CCC-CA-CCC-A-CCC-CCA-CCC, CCC-CA-CCC-AC-CCC-CA-CCC, CCC-CA-CCC-ACC-CCC-A-CCC, CCC-A-CCC-A-CCC-CCA-CCC, CCC-A-CCC-AC-CCC-CA-CCC, and CCC-A-CCC-ACC-CCC-A-CCC). To overcome this limitation, we designed a general pattern for iM formation searching using directed graph traversal process. For one sequence, the C-tracts can be regarded as nodes, and the loops can be defined as edges. All possible C-tracts (C-tract length ≥ 3) are identified as nodes in the first phase, and if the distance between two nodes (loop length) is between one and twelve nucleotides, a directed edge is added between the two nodes. After constructing the directed graph, all possible iM formations and conformations are identified via the traversal of the directed graph from every node. All possible putative iMs are represented with the sub-population containing the first four nodes and three edges of the traversing paths with at least four nodes. To choose the representative iM structures from all possible iM structures, four strategies were introduced (greedy non-overlapping, greedy overlapping, non-greedy non-overlapping, and non-greedy overlapping) maintaining the nomenclature derived from QuadBase2 (12). Overlapping strategy selects an iM representative structure for each iM starting coordinate while the non-overlapping function has no coinciding iM representatives. The greedy strategy maximises the loop length of iM representatives with longest C-tract. For non-greedy strategies, the iM with the most extended C-tract length and the shortest loop length can be selected. One representative iM forming sequence may have many different iM conformations although they share the same sequence content. Two representative iM formations are chosen according to their stability: (A) the structure with minimum standard deviation of loop lengths; (B) the structure with minimum length of the two side loops (iMs have three loops, separated by four C-tracts, and we refer to the loops on both sides as ‘side loops’). The users can also set the configuration to get all putative iM conformations. We called the initial computational pipeline Putative-iM-Searcher (Figure 1A).

### Dataset construction and feature selection for machine learning

We employed Putative-iM-Searcher in high-confident iM-peak regions and interval regions in both Watson and Crick strands in the human reference genome (GRCh38). Putative iMs in high confident iM-peak regions were defined as folded iMs, and unfolded C-rich sequences in interval regions. We used a non-overlapping strategy to avoid bias in the performance estimation of the classification model. Four classification datasets were constructed: (Classification dataset 1) non-overlapping, greedy and conformation A; (Classification dataset 2) non-overlapping, greedy and conformation B; (Classification dataset 3) non-overlapping, non-greedy and conformation A; (Classification dataset 4) non-overlapping, non-greedy and conformation B.

We selected underpinning data with reliable pH_T_ from literature-derived data. We also generated in-house biophysical experimental data, with systematic changes in sequences, for developing regression models. The Putative-iM-Searcher was applied to filter dataset of iM forming sequences with their corresponding pH_T_ values. The known, characterised iMs, their respective sequence pattern and corresponding pH_T_ were used for regression model construction. We filtered iM items with the same putative iM forming sequence but different pH_T_ and combined iM items with the same putative iM forming sequence and pH_T_ to avoid bias. Both our classification model (for iM folding status prediction) and regression model (for iM folding strength estimation) used thirty-three different features: C-tract length, iM length, loop length, middle loop length, longest side loop length, shortest side loop length, sum of two side loops, longest loop length, shortest loop length, A density in iMs, C density in iMs, G density in iMs, T density in iMs, A density in loops, C density in loops, G density in loops, T density in loops, A density in middle loop, C density in middle loop, G density in middle loop, T density in middle loop, A density in longest side loop, C density in longest side loop, G density in longest side loop, T density in longest side loop, A density in shortest side loop, C density in shortest side loop, G density in shortest side loop, T density in shortest side loop, A density in two side loops, C density in two side loops, G density in two side loops, T density in two side loops. For the regression system, the iM folding strength is defined as the pH_T_ after standardization and min-max scaling.

### The imbalanced ensemble learning to predict folded and unfolded i-motifs

A five-fold cross-validation assessment was applied to evaluate the classification performance of the iMs for four datasets via nine widely-used classifiers including Decision Tree (33), Random Forest (34), Balanced Random Forest (35), Naive Bayes (36), Linear Discriminant Analysis (37), Easy Ensemble (38), Balanced Bagging (39,40), Random Undersampling Boosting (RUSBoost) (41) and Extreme Gradient Boosting (XGBoost) algorithms (42). The brief description of these models can be found in Supplementary Table S2. The combination of dataset and model which achieve best performance via area under the receiver operating characteristic curve (AUROC) and balanced accuracy, was used for classification. In the whole dataset, 90% of data was randomly selected and separated into a training & validation set, and the remaining 10% of data was used as the test set. Five-fold cross-validation and grid searching on training & validation set were employed to search for the best hyperparameters and test set was used to evaluate the model's classification performance on accuracy, recall, specificity, and AUROC.

### The regression algorithm to measure the strength of i-motif using ensemble learning

Consistent iM searching and conformation identification strategy with classification dataset was applied in the regression model. A five-fold cross validation assessment was applied to evaluate the regression performance of the iMs based on thirteen widely-used regressors including Decision Tree (33), Random Forest (34), Linear Regression (43), Ridge Regression (44), Lasso Regression (45), Elastic Net Linear Regression (46), Linear Support Vector Regression (47), Radial Basis Function Support Vector Regression (48), K-Nearest Neighbors Regression (KNN) (49), Adaptive Boosting (AdaBoost) (50), Gradient Boosting (51), Extreme Gradient Boosting (XGBoost) (42) and Random Sample Consensus (RANSAC) algorithms (52). The brief description of these models can be found in Supplementary Table S2. In the whole dataset, 80% of data was separated into training & validation set randomly for hyperparameters adjustment by five-fold cross-validation and grid searching, and 20% of data was used to evaluate the regression performance of the model by coefficient of determination (R^2^), root mean squared error (RMSE), and mean absolute error (MAE) (19). The feature importance of the regression model was extracted from the model with ‘importance_type = gain’.

### Implementation

The algorithm was written in Python 3, and machine learning was employed via the Python Scikit-learn package (53), Imbalanced-learn package (54), and XGBoost package (42). The programs and documentation of Putative-iM-Searcher and iM-Seeker are available at Figshare via DOI https://doi.org/10.6084/m9.figshare.24587160.v1.

### Biophysical characterisation of C-rich DNA sequences

The test oligonucleotides were synthesised and reverse phase HPLC purified by Eurogentec (Belgium) and were resuspended in ultra-pure water. The DNA final concentration was confirmed via Nanodrop. Samples were prepared as 10 μM DNA in 10 mM sodium cacodylate (NaCaco) and 100 mM KCl buffer with the range of pH 4–8. The DNA samples were annealed prior to biophysical characterisation by denaturing the DNA for 5 mins at 95°C and allowing to reanneal by slowly cooling down to room temperature, overnight.

The CD spectra of the annealed C-rich sequences were recorded on a JASCO 1,500 spectropolarimeter (JASCO UK Ltd.) under a constant flow of nitrogen. An accumulation of four CD spectra scans was acquired from 200–320 nm at 20°C with a data pitch of 0.5 nm, scanning speed of 200 nm/min with 1 second response time, 1 nm bandwidth, and 200 mdeg sensitivity. The measured DNA samples and buffer at corresponding pH were subtracted before zero correction at 320 nm. The transitional pH (pH_T_) was determined by plotting the measured ellipticity at 288 nm and pH range and the resulting inflection point of the Boltzmann sigmoidal or bi-phase sigmoidal fit using Graphpad Prism (Version 10.1.0.316).

The CD samples at pH 5.5 were diluted in the same buffer to 2.5 μM final DNA concentration. These samples were used to perform UV spectroscopy (V-750ST UV/VIS Spectrophotometer, JASCO UK Ltd.) to obtain the thermal difference spectra (TDS) and determine the melting temperature (*T*_M_), annealing temperature (*T*_A_) and their respective hysteresis (T_H_). For melting/annealing experiments, the absorbance at 295 nm was measured at every 1°C increase/decrease in three cycles of denaturation and reannealing. The cycle begins with 10 mins at 4°C followed by gradual increase of 0.5°C/min to 95°C (melting). Once the final temperature was reached, the samples were kept at 95°C for 10 mins before reversing the process (annealing). The melting and annealing temperatures were determined via the first derivative method of for each measured cycle as previously described (55). The samples were kept at 4°C after the completion of the final reannealing cycle. For the thermal difference spectra (TDS), these samples were used to obtain the absorbance spectrum (230–320 nm). The samples were kept at 4°C for an additional 10 mins before measuring the absorbance spectrum of potentially folded iMs. This was followed by a second absorbance spectrum after 10 mins at 95°C for the unstructured DNA structure. Individual TDS signatures were determined by subtracting both absorbance spectra (unfolded-folded DNA structure), zero correcting at 320 nm, and finally normalisation to the maximum absorbance to 1 as previously described (26).

## Results

### Description of the iM-Seeker framework

iM-Seeker is a computational framework using machine learning to predict the folding status and folding strength of iMs. The outline of the whole iM-Seeker structure is shown in Figure 1. The Putative-iM-Searcher was developed to discover the putative iM forming sequences (Figure 1A). Putative-iM-Searcher constructs a directed graph model and obtains representative conformation from all DNA structure conformations based on the configuration of overlapping & non-overlapping strategy, greedy & non-greedy strategy, and representative-conformation-selection strategy. The Balanced Random Forest classification model and XGBoost regression model were trained on iMab-based genome-wide iM landscape and biophysical experimental justified iM with pH_T_, respectively, for the folding status prediction and folding strength estimation (Figure 1B). The workflow of iM-Seeker after receiving the query sequences is shown in Figure 1C. Putative-iM-Searcher was applied to query sequences to find putative iM forming sequences in the first stage. For each putative iM individual, the Balanced Random Forest classification model will be used to predict the folding status. Next, an estimated folding strength score was calculated by the XGBoost regression model for putative iM individuals.

### iM-Seeker predicts iM structure folding status

Recently published CUT&Tag sequencing data was used to determine empirically the difference between folded iMs and unfolded C-rich sequences (7). This data incorporated among three biological replicates, giving an excellent base for determining iM-formation. Putative iM-forming sequences in the intersected high-confident iM-peak regions from the CUT&Tag sequencing data were defined as folded iMs, while other C-rich sequences in interval regions that did not give iM-peaks were defined as unfolded C-rich sequences. We separated classifications into greedy/non-greedy and non-overlapping classification datasets. Greedy and non-overlapping classification datasets included 8,837 folded iMs and 733,115 unfolded C-rich sequences while 9,641 folded iMs and 755,747 unfolded C-rich sequences were in non-greedy and non-overlapped two datasets.

**Figure 2.**
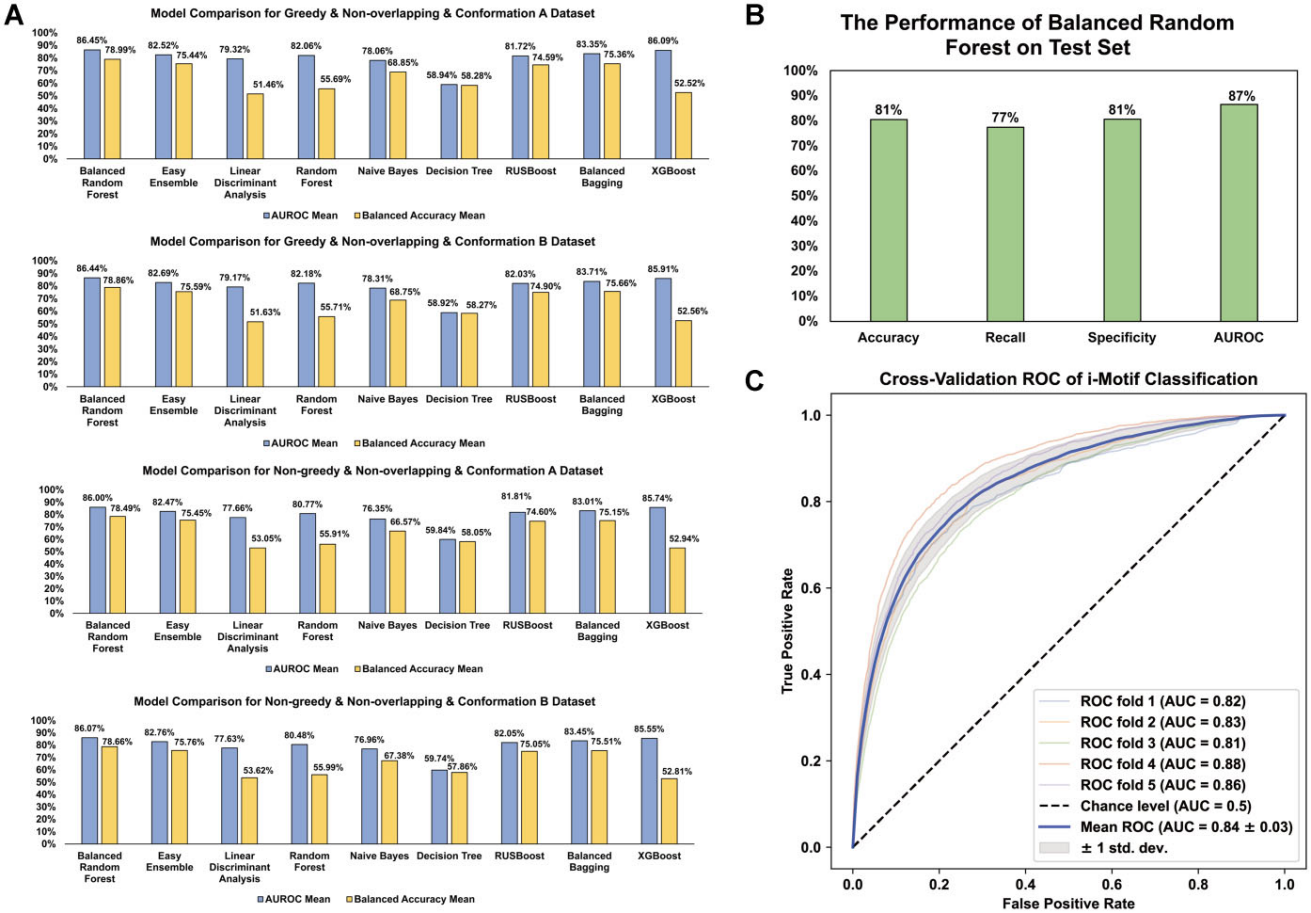
Model selection and performance estimation of classification model. (**A**) The comparison among nine models (Decision Tree, Random Forest, Balanced Random Forest, Naive Bayes, Linear Discriminant Analysis, Easy Ensemble, Balanced Bagging, RUSBoost, and XGBoost) on four classification datasets. AUROC and balanced accuracy show that Balanced Random Forest on greedy & non-overlapping & conformation A dataset has the best performance. (**B**) The performance of Balanced Random Forest classifier on the test set. Accuracy, recall, specificity, and AUROC can reach 81%, 77%, 81% and 87% respectively. (**C**) The ROC curves for classification performance. The Receiver Operating Characteristic (ROC) for the five-fold cross validation is shown. Each fold coloured separately with the AUC score and the mean ROC curve are coloured blue, and the random probability is shown as black dash lines.

Thirty-three features from labelled folded iM and unfolded C-rich sequences were derived. A five-fold cross-validation assessment was applied on nine classifiers on four classification datasets to select the best dataset and model. Considering the mean AUROC score and mean balanced accuracy of five folds, Balanced Random Forest performed best in all four datasets because the balanced learning strategy can better fit our imbalanced datasets. Thus, Balanced Random Forest was selected as the final classifier. Greedy and non-overlapping datasets outperformed the non-greedy and non-overlapping datasets in terms of the mean AUROC score and mean balanced accuracy. Although there is no significant difference between conformation A and B for greedy and non-overlapping datasets, both AUROC and balanced accuracy of conformation A were found to be higher than B (Figure 2A). Thus, the greedy, non-overlapping and conformation A strategy was selected for the final dataset classification task.

The whole dataset was divided into the training & validation set (90%) and test set (10%) because the whole dataset contains ∼740,000 data items, test set with ∼74,000 (10%) data items is enough to test the model performance. The Balanced Random Forest model was optimised by cross-validation and grid search on the training & validation set. We evaluated the model performance on the test set with 81% accuracy, 77% recall, 81% specificity and 87% AUROC score, which show the model can achieve good performance in both folded iMs and unfolded C-rich sequences (Figure 2B). Besides, we assessed the model's generalisation performance through five-fold cross-validation deployed across the entire dataset on AUROC (Figure 2C). The AUROC scores on all 5-fold are all higher than 0.8, which shows the excellent generalisation performance.

### iM-Seeker measures the iM structure stability

The literature-derived data (Supplementary Table S1) and the experimental biophysical data (Supplementary Table S3) were combined to a collection of 206 C-rich DNA sequences with their corresponding pH_T_ values. The comparison of CD spectroscopy, UV spectroscopy, and TDS between representative iM forming sequence and representative non-iM forming sequence shows the reliability of our experiments (Supplementary Figure S1). However, one study contained 196 different sequences which contained only C and T. To avoid bias, these DNA sequences were excluded to avoid misinterpretation and skewing the importance of different nucleotides in the loops. 171 data items were selected as high-confident iM-containing items from 206 items based on criteria including TDS (Supplementary Data Set S1). After filtering data items with the same putative iM but different pH_T_ and combining iM items with the same putative iM and equal pH_T_ from high-confident data items, 120 putative iMs were extracted from the remaining sequence segments using the consistent Putative-iM-Searcher strategies (greedy, non-overlapping, and conformation A) with classification session followed by feature selection (Supplementary Data Set S2). The 120 pH_T_ values standardized and rescaled to range from 0 to 1 via min-max scaling to define iM folding stability.

**Figure 3.**
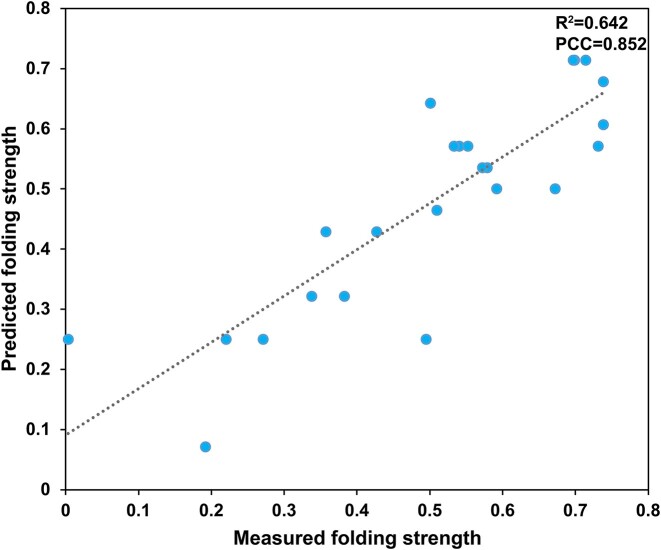
The performance evaluation of the XGBoost regressor on the test set (*n* = 24). The Pearson correlation coefficient (PCC, 0.852, *P*< 1.25×10^−7^) and *R*^2^ (0.642) show a positive correlation between measured and predicted iM folding strength.

A five-fold cross-validation assessment was applied to thirteen regressors on regression datasets to find the model. Considering the mean of three indicators (R^2^, RMSE, and MAE) on five-folds, XGBoost was selected as the final model because of the best performance (Table 1). The whole dataset was divided into training & validation set with 80% data and test set containing the remaining data. After optimization using cross-validation and grid search on training & validation set, the final XGBoost model was applied to the test set to assess the performance. R^2^, RMSE, and MAE can reach 0.642, 0.104 and 0.08, respectively, which shows the model can achieve good performance in estimating the folding strength (Figure 3). The Pearson Correlation Coefficient (PCC) also reveals a strong correlation between measured and predicted folding strength (*P*< 1.25×10^−7^).

**Table 1.** Model comparison of thirteen regressors on iM folding strength estimation

Index	*R* ^2^ mean	Root mean squared error mean	Mean absolute error mean
Linear regression	–0.158	0.195	0.139
Ridge regression	–0.012	0.182	0.132
Lasso regression	–0.027	0.185	0.146
Elastic net linear regression	–0.027	0.185	0.146
Decision tree	–0.002	0.181	0.134
Random forest	0.434	0.138	0.105
Support vector regression	–0.043	0.185	0.130
Radial basis function support vector regression	0.187	0.165	0.120
KNN	0.111	0.173	0.128
AdaBoosting	0.355	0.147	0.113
Gradient boosting	0.379	0.144	0.110
RANSAC	–2.184	0.315	0.222
XGBoost	0.458	0.134	0.103

### Model interpretation provides insights into important features for iM stability

**Figure 4.**
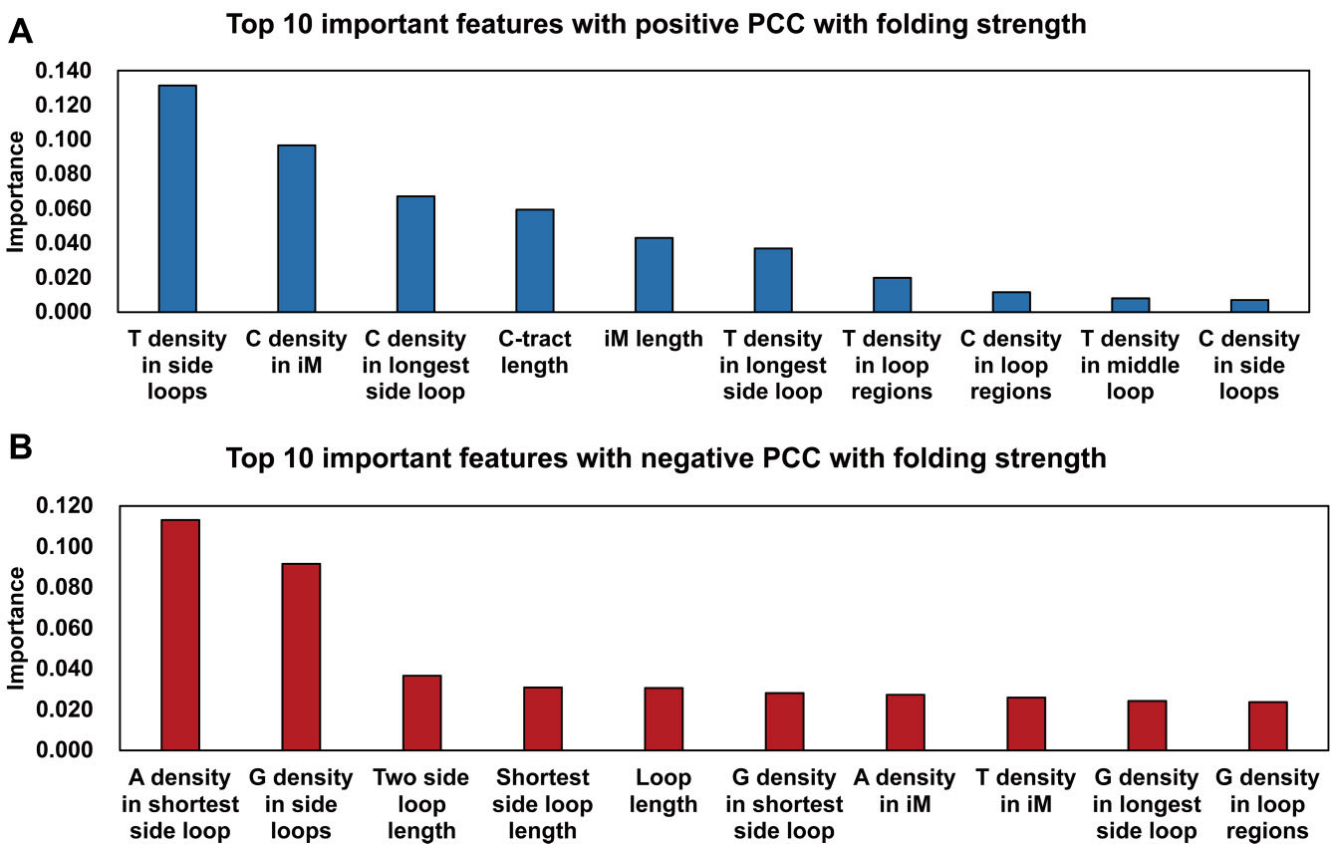
The iM feature importance obtained from the regression model. (**A**) Top 10 important features with positive Pearson correlation coefficient (PCC) with folding stability. (**B**) Top 10 important features with negative Pearson correlation coefficient (PCC) with folding stability.

We investigated the relative importance of the iM features extracted from the regression model. Features with high importance contribute more to the construction of the model and may play a more crucial role in iM formation than features with low importance. We divided the features into two groups based on the Pearson correlation coefficient (PCC): features with positive PCC were assumed to strengthen iM formation (Supplementary Table S4). In contrast, negative-correlated features were supposed to have a negative effect (Supplementary Table S5). In each group, the top 10 critical features are shown in Figure 4. Nucleotide composition affects the stability of iM structures. Stable iMs prefer to contain more C and T, especially T in side loops (Figure 4A). High G density and A density are associated with unstable iMs, especially these two nucleotides in side loops (Figure 4B). In addition, the C-tract length and loop length are two dominant features in all length-relative features. Long C-tract and short loop length can help with iM stability. Previous studies showed that in the same experimental condition, iMs with long C-tracts tend to be more stable than iMs with short C-tracts (56,57).

## Discussion

Unlike the computational prediction of G4 structures, iMs are more complex in terms of what makes them stable (8,29,31,58–61) and it has been difficult to make predictions about iMs in the same way as G4s. Although, putative iMs have a similar sequence pattern to G4s, the stability of the structures has been more difficult to predict, as it has been shown that iMs can tolerate changes in sequence more than G4s (31), but are overall less stable in general. Therefore, iM-specific experimental data is critical to construct accurate computational models for iM prediction and stability. To the best of our knowledge, there are no iM-specific computational tools. Due to the similarity in sequence patterns between G4 and iM, some previous software developed for G4 can be used on putative iM searching and can calculate a numeric value to estimate iM (8,15,23) but there was no iM-specific experimental results which were fed into models to help with model design and training. In this paper, we developed both a putative iM-forming sequence searching tool, Putative-iM-Searcher, and a machine learning approach to prediction of DNA iM folding status and folding strength, iM-Seeker. We considered that the identification of putative iM forming sequences, their folding status and folding strength were three significant parts of iM investigation that could benefit from computational predictions. Putative-iM-Searcher can construct directed graphs based on different configurations, can search all putative iM formations and conformations by graph traversal from input DNA sequences. Users can choose to set parameters including C-tract length, the first loop length, the second loop length, and the third loop length. The representative conformations can be obtained based on overlapping & non-overlapping strategy, greedy & non-greedy strategy, and representative-conformation strategy. Users can choose to obtain all putative iM formations and conformations as well. Based on the detected putative iMs by Putative-iM-Searcher, we used genome-wide CUT&Tag sequencing data and experimental data with pH_T_ from previous studies and our experiments to develop iM-Seeker. Diverse machine learning methods including both traditional machine learning and advanced deep learning have achieved excellent performance in Biology including molecular structure prediction and synthesis (e.g. protein and RNA structure prediction and design), molecular function and interaction investigation (e.g. RNA-protein binding prediction and therapeutic target gene discovery), and so forth (62–67). The wonderful example is the advent of the revolutionary deep learning tools to protein structure like AlphaFold (63) and RoseTTAFold (62). This is the first time a machine learning approach has been applied to classification of this specific DNA structure motif and will significantly improve the accuracy of *in silco* iM prediction. The iMab antibody-based CUT&Tag sequencing data presents the folding status of C-rich sequences and iM-Seeker captures the difference between features in both folded iMs and unfolded C-rich sequences and allows for classification. Another regression model was trained on iM sequences derived from biophysical data, corresponding sequence with pH_T_ to measure the folding strength.

We chose thirty-three features from iM samples for model construction. Considerable proportion of G4 prediction studies adopted the similar strategy to extract features from G4 sequence (some models also used flanking sequences) followed by constructing model via traditional machine learning (19,20). An example is Quadron, which is a gradient boost machine model built on 119 features from G4s and their flanking sequences (20). Although deep learning has shown the power on more complex biological systems to solve hard puzzles, traditional machine learning is still efficient and sensitive for plenty of questions because of their good interpretability and fitness for relatively small dataset (67). iM-Seeker has good performance on both classification and regression tasks modelling on these features via ensemble learning. Generally, the selection of model is determined by the data structure and fitness between model and data. In our dataset, the number of folded iMs (8,837 iMs) is much less than unfolded motifs (733,115 iMs), which can mislead the classifier to overfit the unfolded dataset and classify folded iMs into unfolded category incorrectly. Thus, Balanced Random Forest outperforms other candidate models, because this decision-tree-based ensemble learning model employs an under-sampling strategy to avoid overfitting of unfolded samples. Therefore, both folded samples and unfolded samples have good performance (recall 77%; specificity 81%). XGBoost, another ensemble learning approach which was also used in the G4 classification mission (19), is selected for the estimation of folding strength among thirteen regressors. Although the number of data items for the regression model is limited, the regression part of iM-Seeker can also provide a reliable reference to evaluate the iM strength (*R*^2^ 0.642; RMSE 0.104; MAE 0.08). Previous studies investigated the iM formation features which can influence the iM strength by biophysical characterisation. The length of C-tracts, short loop length and high density of C and T can enhance the formation of iMs because other strong structures can be formed with G and A, which can result in the competition between iM and other structure motifs (5,8,56,57,68,69). Important features extracted from the regression model revealed a consistent result with previous research, which also justifies the reliability of our model. However, the stabilising effect of additional thymines is now quite well documented and consistent with the results observed here (31,70). Also the competition between guanines and cytosines were previously used in G4Hunter (15) as a scoring factor as having the complementary base within the sequences can skew structure formation towards hairpin (31).

iM-Seeker offers users the opportunity for a dedicated iM-searching tool, which is based on machine learning from existing datasets. The approach could be applied to other DNA and RNA structures where there is a wide range of data available, for example to further increase the accuracy of prediction of formation of G4 structures.

## Supplementary Material

gkae092_Supplemental_Files

## Data Availability

The programs and documentation of Putative-iM-Searcher and iM-Seeker are available at Figshare via https://doi.org/10.6084/m9.figshare.24587160.v1.
