# Supplementary material for: Prediction of DNA i-motifs via machine learning
Source: Nucleic Acids Res. 2024 Feb 14;52(5):2188–97. doi: 10.1093/nar/gkae092 (PMC10954440; doi:10.1093/nar/gkae092)
Supplement: gkae092_Supplemental_Files [file gkae092_supplemental_files.zip › supproting information_iM-Seeker.pdf]

Prediction of DNA i-Motifs Via Machine Learning

(Supplementary Information)

Bibo Yang,[1,†] Dilek Guneri,[2,†] Haopeng Yu,[1,†,*] Elisé P. Wright,[3] Wenqian Chen,[2] Zoë A. E. Waller,[2,*] Yiliang Ding[1,*]

[1] Department of Cell and Developmental Biology, John Innes Centre, Norwich Research Park, Norwich, NR4 7UH, UK
[2] School of Pharmacy, University College London, London, WC1N 1AX, UK
[3]Molecular Physiology School of Medicine, and Molecular Medicine Research Group, University of Western Sydney, Campbelltown, NSW 1797, Australia
*To whom correspondence s[2]hould be addressed. Tel: +44 (0)1603 450266; Email: yiliang.ding@jic.ac.uk. Correspondence may also be addressed to haopeng.yu@jic.ac.uk or z.waller@ucl.ac.uk.
†Joint First Authors.

Table S1. Literature-derived data set of i-motif forming DNA sequences and their corresponding transitional pH. The white highlighted sequences were used as input data of Putative-iM-Searcher followed by iM-Seeker.

Sequence Name	Sequence 5' - 3'	pH_T	Ref.
**hTeloC**	TAACCCCCCCCCCCC	6.5	(1)
**C2T3**	CCTTTCCTTTCCTTTCC	6.1	
**C3T3**	CCCTTTCCCTTTCCCTTTCCC	6.7	
**C4T3**	CCCCCTTTCCCCTTTCCCCTTTCCCC	7.1	
**C5T3**	CCCCCTTTCCCCCCCTTTCCCCCCCTTTCCCCCCCTTTCCCCCCCTTTCCCCCCC	7.2	
**C6T3**	CCCCCCTTTCCCCCCTTTCCCCCCTTTCCCCCC	6.8	
**C7T3**	CCCCCCCTTTCCCCCCCTTTCCCCCCCTTTCCCCCCC	7.4	
**C8T3**	CCCCCCCCTTTCCCCCCCCTTTCCCCCCCCTTTCCCCCCCC	7.1	
**C9T3**	CCCCCCCCCTTTCCCCCCCCCTTTCCCCCCCCCTTTCCCCCCCCC	7.3	
**C10T3**	CCCCCCCCCCTTTCCCCCCCCCCTTTCCCCCCCCCCTTTCCCCCCCCCC	7.3	
**C5T1**	CCCCCTCCCCCTCCCCCTCCCCC	6.9	
**C5T2**	CCCCCTTCCCCCTTCCCCCTTCCCCC	7.1	
**C5T4**	CCCCCTTTTCCCCCTTTTCCCCCTTTTCCCCC	6.7	
**AC017019.1**	CCCCCCTCCCCCCCTCCCCCCTCCCCCC	7.1	
**AC018878.3**	CCCCCACCCCCAGCCCCCTTTCCCCC	7.1	
**ATXN2L**	CCCCCCCCCCCCCCCCCCCCCCCC	7	
**CAMK2G**	CCCCCAGGCCCCGCCAGTCCCCCCCCCCGCCCGGCCCCCGGCCCGCCCCC	6.9	
**DAP**	CCCCCGCCCCCGCCCCCGCCCCCGCCCCC	7	
**DRP2**	CCCCCTCTTCCCCTCTCCCCCTCTCCCCCTCTCTCCCTCTTCCCCCTCTCCTTGTCTCCTTCTCTCCCCC	6	
**DUX4L22**	CCCCCGAAACGCGCCCCCCTCCCCCCTCCCCCCTCTCCCCC	7.1	
**GH2**	CCCCCACCCCCACCCCCATCCCCACGCCCCGCCCCCGCCCCC	7.1	
**HIC2**	CCCCCGGGACAGGGACCCTGGCCCCCCCCGACAGGCTGACGCCCACCCCCTCAAACTCTGGTGGACTTACCCCC	6.4	
**HOXC10**	CCCCCACCCCCACCCCCACCCCCC	7.1	
**HOXD10**	CCCCCCCCCCCTCCCCCGCGGCCCCC	7.1	
**JAZF1**	CCCCCCCCGCCCCCGCCCCCGCCCTCCCCCC	7.1	
**MSMO1**	CCCCCGCCCCCGCCCCCGCCCCC	6.7	

Continued.

**NFATC1**	CCCCCGTTTCCCCCGCCAGCCCCAGCGCCCCCCTGCCCGGCCCCC	7.1	(1)
**PIM1**	CCCCCGACGCGCCCCCCAACACACAAACCCCCAGAATCCGCCCCC	7	
**PLCB2**	CCCCCGCCTCTTCTGGAGGCCCCCGCCCCCACCCCC	7	
**QSOX1**	CCCCCGCCCCCGAGCCCCCGCCCCC	7.1	
**RAE1**	CCCCCCGCCCCCCCCGCCCCCCCGCGCCGCCCCCCCCCGCCCCCCGCCCCCGTCCCCCCGCCCCCCCCGCCCCCCCCGCCCCCCGTCCCCCCGCCCCCCCGCCCCCCCGTCCCCCC	6.8	
**RUNX1–1**	CCCCCCCCGCACCCCTTCCCCCGGCCCCCCC	6.7	
**RUNX1–2**	CCCCCCTCCCCCTGCCTCTCCCTCCCCCCTTTCCCC	6.5	
**RUNX1–3**	CCCCCCTTTCCCCTGCCCCCCCTGCCTCCCCC	6.7	
**SHANK1b**	CCCCCCTCCCCCCACCCCCCACCCCCCC	7.1	
**SHANK3**	CCCCCGCCTCCGGCGCAGCCCCCTCGCCACCCCCGCTTCCCTCCCGTCTCAGGCCCCCTCCCCCCGCCGCCCCCGCCCCC	6.6	
**SHANK3b**	CCCCCCGCACCGAGGCCTAGGACTCCCCCCCCCAACCCCGTCACAGCCCCCCAGACCCCCGCCCCGTGGCTCGGCCCCC	6.5	
**SNORD112**	CCCCCCCCCGCCCCCCACCCCCCCACCCCCCCCCCC	7.2	
**SOX1**	CCCCCTGCAGGCCCCCCTGCGCCTCCCCCCCCCCGCCACTGGCGCCTGGCTTCCCCC	6.9	
**STX17**	CCCCCGCCCCCGCCCCCGCCCCGCAGGGCCCCC	7	
**Tandem Repeat (LA16c-OS12.2)**	CCCCCCGTGTCGCTGTTCCCCCCGTGTCGCTGTTCCCCCCGTGTCGCTGTTCCCCCC	6.6	
**TRABD**	CCCCCGCCCCCCCCCCCCCCCCC	6.9	
**WNT7A**	CCCCCGCCCCTCCCTCCTTTCCCCCGTCCCTCCCCCGCCCCCTCCCCC	7.1	
**ZBTB7B**	CCCCCCATCCCTCCCCTCCCTCCCCCCGCCCCTGCCACCCCCCAAACTCCCCCCCCCC	7.1	
**ZFP41**	CCCCCAGCCCCCGCCGACCCCCAGCTCCCGCCTCCGCCGACCCCCAGCCCCC	7	
**ZNF480**	CCCCCGCCCCCGCCCCCGCCCCC	6.7	
**RET20**	CCCCGCCCCGCCCCGCCCCA	7.1	(2)
**c-MYC**	CCCCACCTTCCCCACCCTCCCCACCC	6.6	(3)
**BCL-2**	CCCGCTCCCGCCCCCTTCCTCCCGCGCCCGCCCC	6.6	
**VEGF**	CCCGCCCCCGGCCCGCCCC	5.8	

Continued.

**RET**	CCCGCCCCGCCCCGCCC	6.4	(3)
**Rb**	CCGCCCAAAACCCCCC	5.9	
**h-TELO**	TAACCCTAACCCTAACCCTAACCC	6.5	(4)
**c-MYC**	TCCCCACCTTCCCCACCCTCCCCACCCTCCCCA	6.6	
**PDGFR-β**	GCGTCCACCCTCCCTGCCCCGCCGCCCCCCCTTCTC CCAGC	6.6	
**BCL-2**	CAGCCCCGCTCCCGCCCCCTTCCTCCCGCGCCCGC CCCT	6.6	
**KRAS**	GCCCGGCCCCCGCTCCTCCCCCGCCGGCCCGGCCC GGCCCCCTCCTTCTCCCCG	6.9	
**VEGF**	GACCCCGCCCCCGGCCCGCCCCGG	6	
**Rb**	GCCGCCCAAAACCCCCCG	5.9	
**RET**	CCGCCCCCGCCCCGCCCCGCCCCTA	6.4	
**c-KI-RAS**	GCTCCCTCCCTCCCTCCTTCCCTCCCTCCC	6.6	
**c-KIT**	CCCTCCTCCCAGCGCCCACCCT	6.8	
**HIF-1α**	CGCGCTCCCGCCCCCTCTCCCCTCCCCGCGC	7.2	
**DAP**	CCCCCGCCCCCGCCCCCGCCCCCGCCCCC	7	
**JAZF1**	CCCCCCCCGCCCCCGCCCCCGCCCTCCCCCC	7.1	
**n-MYC**	ACCCCCTGCATCTGCATGCCCCCTCCCACCCCCT	6.5	
**4CT**	CTTCTCCCCACCTTCCCCACCCTCCCCACCCTCCCC	6.9	(5)
**5CT**	CTTCTCCCCACCTTCCCCACCCTCCCCACCCTCCCC ATAAGCGCCCCTCCCG	6.5	
**WT PDGFR-beta NHE Py41**	GCGTCCACCCTCCCTGCCCCGCCGCCCCCCCTTCTC CCAGC	6.6	(6)
**R1 T-to-C mutant Py41**	GCGCCCACCCTCCCTGCCCCGCCGCCCCCCCTTCT CCCAGC	6.6	
**R5 C-to-A mutant Py41**	GCGTCCACCCTCCCTGCCCCGAAGCCCCCCCTTCTC CCAGC	6.4	
**R6 C-to-A mutant Py41**	GCGTCCACCCTCCCTGCCCCGCCGCCACACCTTCTC CCAGC	6.2	
**C3T333**	CCCTTTCCCTTTCCCTTTCCCT	6.6	(7)
**C3T444**	CCCTTTTCCCTTTTCCCTTTTCCCT	6.4	
**C3T555**	CCCTTTTTCCCTTTTTCCCTTTTTCCCT	6.2	
**C3T666**	CCCTTTTTTCCCTTTTTTCCCTTTTTTCCCT	5.8	
**C3T777**	CCCTTTTTTTCCCTTTTTTTCCCTTTTTTTCCCT	5.6	
**C3T888**	CCCTTTTTTTTCCCTTTTTTTTCCCTTTTTTTTCCCT	5.4	
**C3T338**	CCCTTTCCCTTTCCCTTTTTTTTCCC	6.5	
**C3T383**	CCCTTTCCCTTTTTTTTCCCTTTCCC	6.6	
**C3T833**	CCCTTTTTTTTCCCTTTCCCTTTCCC	6.5	
**C3T883**	CCCTTTTTTTTCCCTTTTTTTTCCCTTTCCC	6.2	
**C3T838**	CCCTTTTTTTTCCCTTTCCCTTTTTTTTCCC	6.1	

Continued.

**C3T388**	CCCTTTCCCTTTTTTTTCCCTTTTTTTTTCCC	6.1	(7)
**APE1-4 track**	TACCCACCCCCACCCTGCCCTG	6.1	(8)
**APE1-5 track**	AACCCCCAGGGCTACCCACCCCCACCCTGCCCTG	6.2	
**FEN1**	GTCCCCACTCCACCCACACCAGGTCCCCGCAGGCC CCTGCTCCCTC	6.4	
**MGMT**	CCGCCCCAGCTCCGCCCCCGCGCGCCCCGGCCCCG CCCCCGC	6.7	
**NEIL1**	CGCCCCTCCCTGCGCCCCCCTCCCCCCAC	6.5	
**NEIL2-4 track**	GGCCCGGGGCCCGCCCTCCCTT	5.7	
**NEIL2-5 track**	GGCCCGGGGCCCGCCCTCCCTTCCTGTCCCCTC	6.1	
**NEIL2-6 track**	GGCCCGGGGCCCGCCCTCCCTTCCTGTCCCCTCCC GA	6.3	
**NEIL3-4 track**	GGCCCCGCCCAGGCCCCGCCCAA	5.1	
**NEIL3-5 track**	GGCCCCGCCCAGGCCCCGCCCAAACAGCACCCTA	5.8	
**NTHL1-4 track**	GTCCCGGGCCCTCACCCGCGCCCAC	5.3	
**NTHL1-5 track**	GTCCCGGGCCCTCACCCGCGCCCACTGCAACCCGA	5.7	
**PCNA: sequence 1**	TTCCCTAGCCCCGACCCGAGAGCTCCCTCTCCCGG	5.9	
**PCNA: sequence 2**	CGCCCCGCCCCGCCCCCGTCGCCCTGCCTCCCTG	6.6	
**POLβ**	CGCCCCTCTAGCCCCGCCCCGCCCCGCCCAG	6.5	
**Polη**	GTCCCGACACCCTCTCCCAGCCCCAG	6.2	
**RAD17-Sequence 1**	CGCCCCCAGCCTGCCCCAGCCCAGTCCCTCCCGG	6.2	
**RAD17-Sequence 2**	CCACCCCCCCCCGCCCCCCCCCGGA	6.9	
**RAD21**	TTCCCCACCCCCTCCCCCGACCCTTTTCCCCTCCCC GG	6.5	
**RAD54L**	GGCCCCGCCCCTCCCCCGCCACCCCCGCCCCCGCC CCGCCCCTC	6.5	
**UDG-4 track**	TTCCCAGCCCCCTCCCCCCGACCCCAC	6.7	
**UDG-5 track**	CACCCCTAAGGGGCAGGAACTTTTCTTCCCAGCCCC CTCCCCCGACCCCAC	6.3	

Continued.

**XRCC2**	CGCCCACCGGCGGCCTTGTTCCCATCTCCCTCACTCCCAACCCGG	6	(8)
**XRCC3**	GACCCGCCCCGCCGCCCCGGCCCGGCCCCGC	5.8	
**XRCC5**	TACCCACCCATCCCATCCCTCTTCTCCCTC	6.4	
**hTeloCT**	TCCCTAACCCTAACCCTAACCCAA	6.3	(9)
**ODN2**	CCCGTTGCCCTTTCCCGTTGCCC	7.6	(10)
**ODN3**	CCCGTTGCCCTTTCCCTTTTCCC	7.4	
**ODN6**	CCCGTTGCCCTTTCCCATTACCC	7.1	
**ODN7**	CCCCGTTGCCCTTTCCCCTTTTCCC	7.9	
**dC19**	CCCCCCCCCCCCCCCCCCC	7.5	(11)
**dC18-4132413**	CCCCTCCCTTCCCCTCCC	6.6	
**dC19-4133413**	CCCCTCCCTTTCCCCTCCC	6.4	
**dC19-4141414**	CCCCTCCCCTCCCCTCCCC	6.9	
**dC20-4142414**	CCCCTCCCCTTCCCCTCCCC	6.2	
**LL3**	TCGTTCCGTTTCGTTCCGT	7.8	(12)
**LL4**	TCGTTCCGTTTTCGTTCCGT	7.9	
**LL3rep**	TCGTTCCGTTTTTCGTTCCGTTTTTCGTTCCGTTTTTCGTTCCGT	7.6	
**LL3long**	TCGTTCCGTTTTTCGTTCCGTTTTTTTTCGTTCCGTTTTTCGTTCCGT	7.5	
**TT**	TTCCCTTTCCCTTTCCCTTTCCCTT	6.5	(13)
**AA**	TTCCCTATCCCTTTCCCTATCCCTT	6.2	
**CC**	TTCCCTCTCCCTTTCCCTCTCCCTT	6.4	
**GG**	TTCCCTGTCCCTTTCCCTGTCCCTT	6.2	
**GC**	TTCCCTGTCCCTTTCCCTCTCCCTT	6.3	
**TA**	TTCCCTTTCCCTTTCCCTATCCCTT	6.3	
**CA**	TTCCCTCTCCCTTTCCCTATCCCTT	6.3	
**TG**	TTCCCTTTCCCTTTCCCTGTCCCTT	6.4	
**1C**	TGTCCCCACACCCCTGTCCCCACACCCCTGT	6.5	(14)
**2C**	TGTGCCCACACCCCTGTGCCCACACCCCTGT	5.2	
**3C**	TGTCCTCACACCCCTGTCCTCACACCCCTGT	6.0	
**4C**	TATCCCCACACCCCTATCCCCACACCCCTAT	6.7	
**5C**	TATCCACACACCCCTATCCACACACCCCTAT	6.8/5.9	
**6C**	TGTCCCCAGACCCCTGTCCCCAGACCCCTGT	6.2	
**7C**	TGTCCTCAGACCCCTGTCCTCAGACCCCTGT	5.4	
**8C**	TGTCCCCGGACCCCTGTCCCCGGACCCCTGT	5.1	
**9C**	TGTCCCCAGGACCCCTGTCCCCAGGACCCCTGT	5.5	
**10C**	TGTCCCCAGGACCCTGTCCCCAGGACCCTGT	4.7	

Continued.

**11C**	TGTCCCCGGGACCCCTGTCCCCGGGACCCCTGT	4.7	(14)
**AC017019.1**	CCCCCCTCCCCCCCTCCCCCCTCCCCCC	7.0	(15)
**DAP**	CCCCCGCCCCCGCCCCCGCCCCCGCCCCC	5.9/7.1	
**PIM1**	CCCCCGACGCGCCCCCCAACACACAAACCCCCAGAATCCGCCCCC	6.6	
**ZBTB7B**	CCCCCCATCCCTCCCCTCCCTCCCCCCGCCCCTGCCACCCCCCAAACTCCCCCCCCCC	6.7	
**DUX4L22**	CCCCCGAAACGCGCCCCCCTCCCCCCTCCCCCCTCTCCCCC	6.9	
**DUX4L22MUT**	CCTCCGAAACGCGCCTTCCTCCTTCCTCCTTCCTCTCCTCC	6.3	
**SNORD112**	CCCCCCCCCGCCCCCCACCCCCCCACCCCCCCCCCC	7.1	
**SNORD112 MUT**	CCTCCTTCCGCCTTCCACCTTCTCACCTCCTCCTCC	5.5/6.6	
**MYCPu22rev comp**	TTACCCACCCTACCCACCCTCA	5.9	
**GGGT rev comp**	ACCCACCCACCCACCC	5.8	

Table S2. Overview of machine learning algorithms tested in iM-Seeker.

**Algorithms**	**Tested categories**	**Description**	**Ref.**
Decision Tree	Folding status prediction<br>Folding strength estimation	A non-parametric supervised model by building a tree-like statistic model.	(16)
Random Forest	Folding status prediction<br>Folding strength estimation	Ensemble learning model integrated ensemble of decision trees.	(17)
Balanced Random Forest	Folding status prediction	Adjusted Random Forest with under-sampling strategy to avoid overfitting imbalanced labeled dataset.	(18)
Naive Bayes	Folding status prediction	Supervised model based on Bayes theory to assume the conditional independence of every pair of variables given a label.	(19)
Linear Discriminant Analysis	Folding status prediction	Supervised model based on Fisher's Linear Discriminant to project data point to lower dimensions to maximize the difference between labels.	(20)
Easy Ensemble	Folding status prediction	Ensemble learning model integrated ensemble of AdaBoost learners trained on different balanced samples from random under-sampling to avoid overfitting imbalanced labeled dataset.	(21)
Balanced Bagging	Folding status prediction	Ensemble learning model using bagging strategy to integrate ensemble of weak learners trained on different balanced samples from random under-sampling to avoid overfitting imbalanced labeled dataset.	(22,23)
Random Undersampling Boosting	Folding status prediction	Adjusted Adaptive Boosting ensemble learning with random under-sampling to avoid overfitting imbalanced labeled dataset.	(24)
Extreme Gradient Boosting	Folding status prediction<br>Folding strength estimation	Adjusted Gradient Boosting integrated ensemble of weak learners. Compared with Gradient	(25)

		Boosting, Extreme Gradient Boosting can be better in both engineering perspective and performance.	
Linear Regression	Folding strength estimation	Supervised model to investigate the linear relationship between response variable and explanatory variables.	(26)
Ridge Regression	Folding strength estimation	Adjusted linear regression with L2 regularization to reduce training data overfitting.	(27)
Lasso Regression	Folding strength estimation	Adjusted linear regression with L1 regularization to reduce training data overfitting.	(28)
Elastic Net Linear Regression	Folding strength estimation	Adjusted linear regression with both L1 and L2 regularization to reduce training data overfitting.	(29)
Linear Support Vector Regression	Folding strength estimation	Supervised model based on trying to find a hyperplane in searching space to separate the labels via linear kernel.	(30)
Radial Basis Function Support Vector Regression	Folding strength estimation	Supervised model based on trying to find a hyperplane in searching space to separate the labels via Radial Basis Function kernel.	(31)
K-Nearest Neighbors Regression	Folding strength estimation	A non-parametric supervised model using K-Nearest Neighbors to predict the outcomes.	(32)
Adaptive Boosting	Folding strength estimation	Ensemble learning model integrated weak learners using adaptive boosting strategy.	(33)
Gradient Boosting	Folding strength estimation	Ensemble learning model integrated weak learners using gradient boosting strategy.	(34)
Random Sample Consensus	Folding strength estimation	Supervised model using Random Sample Consensus to estimate the model parameters.	(35)

Table S3. Biophysical analysis of c-rich sequences with indicated location of nucleotide replacement or deletion (Δ) of the predominant ILPR (ACA) sequence. Variations of hTeloC and some known iM forming sequences are included in the experimental set up. The white highlighted sequences were used as input data of Putative-iM-Searcher followed by iM-Seeker. Thermodynamics data are presented as Mean ± SD (n=3).

**Name**	**Sequence 5' - 3'**	**Thermodynamics UV spectroscopy (295 nm)**			**CD Spectroscopy**	**TDS**
		T_M (°C)	T_A (°C)	ΔH (°C)	pH_T	Structure
**ACA**	TGTCCCCACACCCCTGTCCCCACACCCCTGT	55 ± 1.7	52 ± 0.9	3 ± 1.0	6.6	iM
**ACA(C1/9=T)**	TGT**T**CCCACACCCCTGT**T**CCCACACCCCTGT	50 ± 0.3	48 ± 0.0	2 ± 0.2	6.4	iM
**ACA (C2/10=T)**	TGTC**T**CCACACCCCTGTC**T**CCACACCCCTGT	40 ± 0.5	38 ± 0.6	2 ± 0.4	5.7 / 6.5	iM
**ACA(C3/11=T)**	TGTCC**T**CACACCCCTGTCC**T**CACACCCCTGT	40 ± 0.3	37 ± 1.0	3 ± 1.2	6.0	iM
**ACA (C4/12=T)**	TGTCCC**T**ACACCCCTGTCCC**T**ACACCCCTGT	48 ± 0.3	45 ± 0.4	3 ± 0.7	5.7 / 6.5	iM
**ACA (C5/13=T)**	TGTCCCCACA**T**CCCTGTCCCCACA**T**CCCTGT	53 ± 0.6	50 ± 0.6	3 ± 0.6	5.4 / 6.7	iM
**ACA (C6/14=T)**	TGTCCCCACAC**T**CCTGTCCCCACAC**T**CCTGT	44 ± 0.0	41 ± 0.5	2 ± 0.5	5.9 / 6.7	iM
**ACA (C7/15=T)**	TGTCCCCACACC**T**CTGTCCCCACACC**T**CTGT	37 ± 0.6	35 ± 0.6	2 ± 0.0	5.9	iM
**ACA (C8/16=T)**	TGTCCCCACACCC**T**TGTCCCCACACCC**T**TGT	44 ± 0.0	43 ± 0.6	1 ± 0.6	6.0	iM
**ACA (C1/5/9/13=T)**	TGT**T**CCCACA**T**CCCTGT**T**CCCACA**T**CCCTGT	49 ± 0.6	45 ± 0.1	4 ± 0.6	6.3	iM
**ACA (C2/6/10/14=T)**	TGTC**T**CCACAC**T**CCTGTC**T**CCACAC**T**CCTGT	32 ± 0.4	28 ± 0.5	4 ± 0.3	6.0	iM
**ACA (C3/7/11/15=T)**	TGTCC**T**CACACC**T**CTGTCC**T**CACACC**T**CTGT	29 ± 0.3	27 ± 0.5	2 ± 0.4	5.6	iM
**ACA (C4/8/12/16=T)**	TGTCCC**T**ACACCC**T**TGTCCC**T**ACACCC**T**TGT	44 ± 0.4	41 ± 0.2	3 ± 0.3	6.1	iM
**ACA (C1-odd)**	TGT**T**C**T**CACA**T**C**T**CTGT**T**C**T**CACA**T**C**T**CTGT	ND	ND	ND	ND	B-DNA
**ACA (C2-even)**	TGTC**T**C**T**ACAC**T**C**T**TGTC**T**C**T**ACAC**T**C**T**TGT	ND	ND	ND	ND	B-DNA
**ACA (C1/3/7/9=T)**	TGT**T**C**T**CACACCCCTGT**T**C**T**CACACCCCTGT	31 ± 0.0	29 ± 0.2	2 ± 0.2	5.9	iM

Continued.

Name	Sequence 5' - 3'	Thermodynamics UV spectroscopy (295 nm)			CD Spectroscopy	TDS
		T_M (°C)	T_A (°C)	ΔH (°C)	pH_T	Structure
**ACA (C6/8/14/16=T)**	TGTCCCCACAC**T**C**T**TGTCCCCACAC**T**C**T**TGT	30 ± 0.5	29 ± 0.0	2 ± 0.5	5.7	iM
**ACA (C2/6/10/14=T)**	TGTC**T**CCACAC**T**CCTGTC**T**CCACAC**T**CCTGT	32 ± 0.4	28 ± 0.5	4 ± 0.3	6.0	iM
**ACA (G=T)**	TTTCCCCACACCCCTTTCCCCACACCCCTTT	55 ± 0.0	54 ± 0.6	1 ± 0.6	6.7	iM
**ACA (A=T)**	TGTCCCCTCTCCCCTGTCCCCTCTCCCCTGT	61 ± 0.0	56 ± 0.0	2 ± 0.0	6.9	iM
**ACA=TTT**	TGTCCCCTTTCCCCTGTCCCCTTTCCCCTGT	60 ± 0.3	58 ± 0.0	2 ± 0.3	6.8	iM
**ACA (C1/2/9/10=d)**	TGT**ΔΔ**CCACACCCCTGT**ΔΔ**CCACACCCCTGT	36 ± 0.3	34 ± 0.1	2 ± 0.3	5.7	iM
**ACA (C5/6/9/10=d)**	TGTCCCCACA**ΔΔ**CCTGT**ΔΔ**CCACACCCCTGT	ND	ND	ND	5.5	iM
**ACA (C_3)**	TGTCCC**Δ**ACACCC**Δ**TGTCCC**Δ**ACACCC**Δ**TGT	40 ± 0.4	38 ± 0.1	2 ± 0.4	6.0	iM
**ACA (C2)**	TGTCC**ΔΔ**ACACC**ΔΔ**TGTCC**ΔΔ**ACACC**ΔΔ**TGT	36 ± 0.2	34 ± 0.1	2 ± 0.3	ND	Z-DNA
**ACA (C1/9=d)**	TGTCCC**Δ**ACACCCCTGT**Δ**CCCACACCCCTGT	25 ± 1.0<br>49 ± 0.6	18 ± 0.6<br>47 ± 0.6	7 ± 0.6<br>2 ± 0.6	6.9<br>6.0	iM
**ACA (C5/13=d)**	TGTCCCCACA**Δ**CCCTGTCCCCACA**Δ**CCCTGT	46 ± 0.0	44 ± 0.0	2 ± 0.0	6.3	iM
**ACA (G=d)**	T**Δ**TCCCCACACCCCT**Δ**TCCCCACACCCCT**Δ**T	54 ± 0.2	52 ± 0.0	2 ± 0.2	6.7	iM
**ACA (T1/3/5=d)**	**Δ**GTCCCCACACCCC**Δ**GTCCCCACACCCC**Δ**GT	58 ± 0.1	55 ± 0.5	2 ± 0.6	6.9	iM
**ACA (T2/4/6=d)**	TG**Δ**CCCCACACCCCTG**Δ**CCCCACACCCCTG**Δ**	53 ± 0.0	51 ± 0.3	2 ± 0.3	6.5	iM
**ACA (T=d)**	**Δ**G**Δ**CCCCACACCCCGCCCCACACCCC**Δ**G**Δ**	54 ± 0.2	52 ± 0.0	2 ± 0.2	6.7	iM
**ACA (A1/3=d)**	TGTCCCC**Δ**CACCCCTGTCCCC**Δ**CACCCCTGT	58 ± 0.6	55 ± 0.0	2 ± 0.6	6.7	iM

Continued.

Name	Sequence 5' - 3'	Thermodynamics UV spectroscopy (295 nm)			CD Spectroscopy	TDS
		T_M (°C)	T_A (°C)	ΔH (°C)	pH_T	Structure
**ACA (A2/4=d)**	TGTCCCCAC**Δ**CCCCTGTCCCCAC**Δ**CCCCTGT	59 ± 0.0	57 ± 0.0	2 ± 0.0	6.7	iM
**ACA (A=d)**	TGTCCCC**Δ**C**Δ**CCCCTGTCCCC**Δ**C**Δ**CCCCTGT	58 ± 0.1	56 ± 0.0	2 ± 0.2	5.0 / 6.9	Mixed
**ACA (L-C=d)**	TGTCCCCA**Δ**ACCCCTGTCCCCA**Δ**ACCCCTGT	53 ± 0.1	51 ± 0.0	2 ± 0.1	6.6	iM
**ACA(C9=G)**	TGTCCCCACACCCCTGT**G**CCCACACCCCTGT	49 ± 0.0	47 ± 0.0	2 ± 0.0	6.0	Mixed
**ACA(C1=G)**	TGT**G**CCCACACCCCTGTCCCCACACCCCTGT	49 ± 0.6	47 ± 0.9	2 ± 1.1	6.0	iM
**AGG**	TGTCCCCAGGCCCCTGTCCCCAGGCCCCTGT	53 ± 0.3	50 ± 0.6	3 ± 0.5	5.8	iM
**AGG (C5=A)**	TGTCCCCAGGACCCTGTCCCCAGGCCCCTGT	35 ± 1.3<br>41 ± 0.2<br>62 ± 2.1	26 ± 5.4<br>38 ± 0.6<br>45 ± 1.1	10 ± 1.3<br>15 ± 3.3<br>15 ± 0.2	5.0 / 5.8	Mixed
**AGG (C13=A)**	TGTCCCCAGGCCCCTGTCCCCAGGACCCTGT	36 ± 2.7<br>56 ± 3.2	23 ± 3.7<br>44 ± 3.5	12 ± 3.2<br>12 ± 2.7	5.5	Mixed
**ACA(C1/9=A)**	TGTACCCACACCCCTGTACCCACACCCCTGT	48 ± 0.0	46 ± 0.8	3 ± 0.8	6.0	iM
**(TAT)ACA(C3/11=A)**	TATCCACACACCCCTATCCACACACCCCTAT	40 ± 0.6	38 ± 0.8	2 ± 1.3	5.8 / 6.7	Mixed
**(TAT)ACA(C11=A)**	TATCCCCACACCCCTATCCACACACCCCTAT	43 ± 0.7	40 ± 0.0	2 ± 0.6	5.8 / 6.2	iM
**(TAT)ACA(C3=A)**	TATCCACACACCCCTATCCCCACACCCCTAT	42 ± 0.6	40 ± 0.0	2 ± 0.6	6.0	iM
**(TAT)ACA(C3/11=G)**	TATCCGCACACCCCTATCCGCACACCCCTAT	42 ± 0.7	40 ± 0.0	3 ± 0.9	5.6 / 6.2	iM
**(TAT)ACA(C3/11=T)**	TATCCTCACACCCCTATCCTCACACCCCTAT	42 ± 0.0	40 ± 0.6	2 ± 0.6	6.0	iM
**postILPR1**	CCCCTGCCGCCTGGCCC	ND	ND	ND	5.1	Mixed
**postILPR2**	CCCCCCCACCCCAGGCCC	51 ± 0.2	44 ± 0.5	8 ± 0.6	6.25	iM

Continued.

Name	Sequence 5' - 3'	Thermodynamics UV spectroscopy (295 nm)			CD Spectroscopy	TDS
		T_M (°C)	T_A (°C)	ΔH (°C)	pH_T	Structure
**preILPRC**	CCCCTCCCTCACTCCCACTCTCCCACCCCCA CCACC	50 ± 0.4	48 ± 0.2	2 ± 0.4	6.3	iM
**CHairpin 2:2**	CCTACCTACCTACCTTTTCCTACCTACCTACC	31 ± 0.3	29 ± 0.0	2 ± 0.3	6.0	iM
**CHairpinhTeloC TTA/AAT**	AATCCCAATCCCATTCCCATTCCC	46 ± 0.4	43 ± 0.4	3 ± 0.3	6.5	iM
**hTeloC**	TAACCCTAACCCTAACCCTAACCC	47 ± 0.2	43 ± 0.2	4 ± 0.3	6.5	iM
**hTeloCTGG**	TGGCCCTGGCCCTGGCCCTGGCCC	ND	ND	ND	5.8	Hairpin
**NRF2C**	CCCTCCCGCCCTTGCTCCCTTCCC	46 ± 0.2	43 ± 0.4	2 ± 0.3	6.7	iM

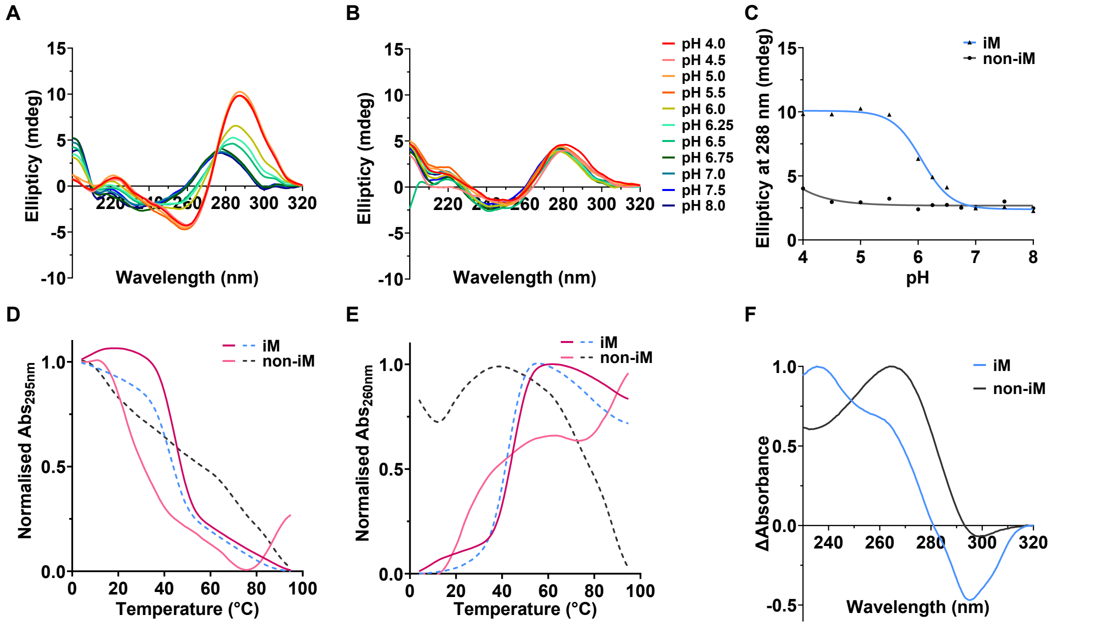

Figure S1. Representative biophysical characterisation of two C-rich DNA sequences. The pH-sensitivity was determined via CD spectroscopy of 10 uM DNA in 10 mM NaCaco 100 mM KCl buffer with a pH range from 4-8 in an iM forming sequence (A; 5'-TGTCCCCACACCCTTGTCCCCACACCCTTGT-3', ACA (C8/16=T) in Table S2) and non-iM forming sequence (B; 5'-TGTTCTCACATCTCTGTTCTCACATCTCTGT-3', ACA (C1-odd) in Table S2). The resulting ellipticity at 288 nm at corresponding pH presents the transitional pH at infliction point of the curve (C). The melting (solid) and annealing (dashed) temperatures for both DNA structures measured at 295 nm (D) and 260 nm (E). The TDS signature profile further confirms iM formation (blue) and non-iM forming sequence (dark grey) (F).

Table S4. Important features with positive Pearson Correlation Coefficient (PCC) between folding stability and feature importance.

**Feature**	**Feature Importance**	**Pearson Correlation Coefficient (PCC)**	**P value of PCC**
T density in side loops	0.131	0.174	0.058
C density in iM	0.097	0.391	0.000
C density in longest side loop	0.067	0.138	0.133
C-tract length	0.059	0.457	0.000
iM length	0.043	0.199	0.030
T density in longest side loop	0.037	0.155	0.091
T density in loop regions	0.020	0.138	0.133
C density in loop regions	0.011	0.124	0.178
T density in middle loop	0.008	0.058	0.530
C density in side loops	0.007	0.119	0.194
C density in middle loop	0.003	0.050	0.586
T density in shortest side loop	0.003	0.152	0.098
C density in shortest side loop	0.003	0.051	0.582

Table S5. Important features with negative Pearson Correlation Coefficient (PCC) between folding stability and feature importance.

Feature	Feature Importance	Pearson Correlation Coefficient (PCC)	P value of PCC
A density in shortest side loop	0.113	-0.149	0.103
G density in side loops	0.091	-0.138	0.133
Two side loop length	0.037	-0.084	0.361
Shortest side loop length	0.031	-0.108	0.242
Loop length	0.030	-0.103	0.265
G density in shortest side loop	0.028	-0.091	0.323
A density in iM	0.027	-0.295	0.001
T density in iM	0.026	-0.044	0.635
G density in longest side loop	0.024	-0.141	0.125
G density in loop regions	0.024	-0.155	0.091
G density in iM	0.021	-0.235	0.010
A density in loop regions	0.020	-0.212	0.020
G density in middle loop	0.011	-0.075	0.418
longest side loop length	0.011	-0.053	0.568
Middle loop length	0.007	-0.119	0.195
A density in side loops	0.004	-0.228	0.012
Shortest loop length	0.003	-0.116	0.209
A density in middle loop	0.002	-0.078	0.394
Longest loop length	0.001	-0.075	0.416
A density in longest side loop	0.000	-0.228	0.012

References

1. Wright, E.P., Huppert, J.L. and Waller, Z.A. (2017) Identification of multiple genomic DNA sequences which form i-motif structures at neutral pH. *Nucleic acids research*, **45**, 2951-2959.
2. Bielecka, P., Dembska, A. and Juskowiak, B. (2019) Monitoring of pH using an i-motif-forming sequence containing a fluorescent cytosine analogue, tC. *Molecules*, **24**, 952.
3. Brooks, T.A., Kendrick, S. and Hurley, L. (2010) Making sense of G‐quadruplex and i‐motif functions in oncogene promoters. *The FEBS journal*, **277**, 3459-3469.
4. Debnath, M., Fatma, K. and Dash, J. (2019) Chemical regulation of DNA i‐motifs for nanobiotechnology and therapeutics. *Angewandte Chemie*, **131**, 2968-2983.
5. Sutherland, C., Cui, Y., Mao, H. and Hurley, L.H. (2016) A mechanosensor mechanism controls the G-quadruplex/i-motif molecular switch in the MYC promoter NHE III1. *Journal of the American Chemical Society*, **138**, 14138-14151.
6. Brown, R.V., Wang, T., Chappeta, V.R., Wu, G., Onel, B., Chawla, R., Quijada, H., Camp, S.M., Chiang, E.T. and Lassiter, Q.R. (2017) The consequences of overlapping G-quadruplexes and i-motifs in the platelet-derived growth factor receptor β core promoter nuclease hypersensitive element can explain the unexpected effects of mutations and provide opportunities for selective targeting of both structures by small molecules to downregulate gene expression. *Journal of the American Chemical Society*, **139**, 7456-7475.
7. Gurung, S.P., Schwarz, C., Hall, J.P., Cardin, C.J. and Brazier, J.A. (2015) The importance of loop length on the stability of i-motif structures. *Chemical Communications*, **51**, 5630-5632.
8. Rogers, R.A., Fleming, A.M. and Burrows, C.J. (2018) Rapid screen of potential i-motif forming sequences in DNA repair gene promoters. *ACS omega*, **3**, 9630-9635.
9. Wright, E.P., Abdelhamid, M.A., Ehiabor, M.O., Grigg, M.C., Irving, K., Smith, N.M. and Waller, Z.A.E. (2020) Epigenetic modification of cytosines fine tunes the stability of i-motif DNA. *Nucleic Acids Research*, **48**, 55-62.
10. Fujii, T. and Sugimoto, N. (2015) Loop nucleotides impact the stability of intrastrand i-motif structures at neutral pH. *Physical Chemistry Chemical Physics*, **17**, 16719-16722.
11. Fleming, A.M., Ding, Y., Rogers, R.A., Zhu, J., Zhu, J., Burton, A.D., Carlisle, C.B. and Burrows, C.J. (2017) 4 n − 1 is a "sweet spot" in DNA i-motif folding of 2′-deoxycytidine homopolymers. *Journal of the American Chemical Society*, **139**, 4682-4689.
12. Mir, B., Serrano, I., Buitrago, D., Orozco, M., Escaja, N. and González, C. (2017) Prevalent sequences in the human genome can form mini i-motif structures at physiological pH. *Journal of the American Chemical Society*, **139**, 13985-13988.
13. Benabou, S., Garavís, M., Lyonnais, S., Eritja, R., González, C. and Gargallo, R. (2016) Understanding the effect of the nature of the nucleobase in the loops on the stability of the i-motif structure. *Physical Chemistry Chemical Physics*, **18**, 7997-8004.
14. Guneri, D., Alexandrou, E., El Omari, K., Dvorakova, Z., Chikhale, R.V., Pike, D., Waudby, C.A., Morris, C.J., Haider, S. and Parkinson, G.N. (2023) Structural Insights into Regulation of Insulin Expression Involving i-Motif DNA Structures in the Insulin-Linked Polymorphic Region. *bioRxiv*, 2023.2006. 2001.543149.
15. Williams, S.L., Casas-Delucchi, C.S., Raguseo, F., Guneri, D., Li, Y., Minamino, M., Fletcher, E.E., Yeeles, J.T., Keyser, U.F., Waller, Z.A. *et al.* (2023) Replication-induced DNA secondary structures drive fork uncoupling and breakage. *The EMBO Journal*, **42**, e114334.

16. Kingsford, C. and Salzberg, S.L. (2008) What are decision trees? *Nature biotechnology*, **26**, 1011-1013.
17. Breiman, L. (2001) Random forests. *Machine learning*, **45**, 5-32.
18. Chen, C., Liaw, A. and Breiman, L. (2004) Using random forest to learn imbalanced data. *University of California, Berkeley*, **110**, 24.
19. Webb, G.I., Keogh, E. and Miikkulainen, R. (2010) Naïve Bayes. *Encyclopedia of machine learning*, **15**, 713-714.
20. Balakrishnama, S. and Ganapathiraju, A. (1998) Linear discriminant analysis-a brief tutorial. *Institute for Signal and information Processing*, **18**, 1-8.
21. Liu, X.-Y., Wu, J. and Zhou, Z.-H. (2008) Exploratory undersampling for class-imbalance learning. *IEEE Transactions on Systems, Man, and Cybernetics, Part B (Cybernetics)*, **39**, 539-550.
22. Maclin, R. and Opitz, D. (1997) An empirical evaluation of bagging and boosting. *AAAI/IAAI*, **1997**, 546-551.
23. Hido, S., Kashima, H. and Takahashi, Y. (2009) Roughly balanced bagging for imbalanced data. *Statistical Analysis and Data Mining: The ASA Data Science Journal*, **2**, 412-426.
24. Seiffert, C., Khoshgoftaar, T.M., Van Hulse, J. and Napolitano, A. (2009) RUSBoost: A hybrid approach to alleviating class imbalance. *IEEE transactions on systems, man, and cybernetics-part A: systems and humans*, **40**, 185-197.
25. Chen, T. and Guestrin, C. (2016) XGBoost: A scalable tree boosting system. *Proceedings of the 22nd acm sigkdd international conference on knowledge discovery and data mining*, 785-794.
26. Su, X., Yan, X. and Tsai, C.L. (2012) Linear regression. *Wiley Interdisciplinary Reviews: Computational Statistics*, **4**, 275-294.
27. McDonald, G.C. (2009) Ridge regression. *Wiley Interdisciplinary Reviews: Computational Statistics*, **1**, 93-100.
28. Tibshirani, R. (1996) Regression shrinkage and selection via the lasso. *Journal of the Royal Statistical Society Series B: Statistical Methodology*, **58**, 267-288.
29. Zou, H. and Hastie, T. (2005) Regularization and variable selection via the elastic net. *Journal of the Royal Statistical Society Series B: Statistical Methodology*, **67**, 301-320.
30. Awad, M., Khanna, R., Awad, M. and Khanna, R. (2015) Support vector regression. *Efficient learning machines: Theories, concepts, and applications for engineers and system designers*, 67-80.
31. Wang, J., Chen, Q. and Chen, Y. (2004) RBF kernel based support vector machine with universal approximation and its application. *International symposium on neural networks*, 512-517.
32. Altman, N.S. (1992) An introduction to kernel and nearest-neighbor nonparametric regression. *The American Statistician*, **46**, 175-185.
33. Freund, Y. and Schapire, R.E. (1997) A decision-theoretic generalization of on-line learning and an application to boosting. *Journal of computer and system sciences*, **55**, 119-139.
34. Friedman, J.H. (2001) Greedy function approximation: a gradient boosting machine. *Annals of statistics*, 1189-1232.
35. Fischler, M.A. and Bolles, R.C. (1981) Random sample consensus: a paradigm for model fitting with applications to image analysis and automated cartography. *Communications of the ACM*, **24**, 381-395.
